# AI-Driven Breast Cancer Nuclei Segmentation, Classification, and Scoring in PR-IHC Images

**DOI:** 10.3390/diagnostics16091295

**Published:** 2026-04-26

**Authors:** Hasanul Bannah, Mohammad Faizal Ahmad Fauzi, Sarina Mansor, Md Serajun Nabi, Md Sabbir Hossen, Seow Fan Chiew, Phaik Leng Cheah, Lai Meng Looi

**Affiliations:** 1 Faculty of Artificial Intelligence and Engineering, Multimedia University, Cyberjaya 63100, Malaysia; mrbannah@gmail.com (H.B.);; 2 School of Digital Health, KPJ Healthcare University, Nilai 71800, Malaysia; 3 Centre for Image and Vision Computing (CIVC), COE for Artificial Intelligence, Multimedia University (MMU), Cyberjaya 63100, Malaysia; 4 Department of Pathology, University Malaya Medical Centre (UMMC), Kuala Lumpur 59100, Malaysia

**Keywords:** Allred scoring, progesterone receptor, immunohistochemistry, nuclei segmentation, nuclei classification, breast carcinoma, deep learning, digital pathology

## Abstract

**Background:** Progesterone receptor (PR) status plays an important role in guiding hormone therapy decisions in breast cancer. In current practice, PR expression is assessed manually from immunohistochemistry (IHC) slides, which can be time-consuming and may vary between pathologists. This study aims to develop an automated and interpretable framework for PR-IHC analysis to improve consistency and efficiency. **Methods:** In this work, we developed an AI-assisted pipeline that combines nuclei segmentation, classification, and scoring for PR-IHC images. A fine-tuned Cellpose model was used to segment individual nuclei. The segmented nuclei were then analyzed using a DAB intensity-based approach to classify them into four categories: negative, weak, moderate, and strong. These results were further combined to generate Allred scores. The system was evaluated on 250 PR-IHC images with annotations provided by expert pathologists. **Results:** The framework achieved strong segmentation performance (F1-score = 0.85, IoU = 0.74) and high classification accuracy (macro F1-score = 0.95). The method also performed well when applied to ER-IHC images without additional retraining. **Conclusions:** The proposed framework provides a reliable and interpretable approach for automated PR-IHC scoring. It helps reduce manual effort, improves consistency in evaluation, and shows potential for practical use in digital pathology settings.

## 1. Introduction

Breast cancer is the most frequently diagnosed cancer among women in the world and continues to be one of the leading causes of cancer death [1]. Mammography [2], ultrasound [3], and magnetic resonance imaging (MRI) [4] are commonly used for initial detection; however, definitive diagnosis and prognosis rely on histopathological analysis of biopsy or surgical tissue samples. The evaluation of hormone receptor status is important for treatment planning, particularly regarding the presence or absence of estrogen receptors (ERs), progesterone receptors (PRs), and human epidermal growth factor receptor 2 (HER2) [5,6]. These biomarkers provide essential information for tumor characterization and treatment selection. The expression of progesterone receptors (PRs) is important along with estrogen receptors (ERs) in clinical practice, as patients with hormone receptor-positive tumors are likely to benefit from endocrine therapy [7].

Predictive immunohistochemistry (IHC) is the standard technique for evaluating hormone receptor expression in breast cancer, enabling assessment of PR status in formalin-fixed, paraffin-embedded (FFPE) tissue sections [5,8]. The hormone receptor profile is essential for determining appropriate treatment strategies. Hormonal therapies have been shown to improve outcomes in PR-positive tumors, while PR-negative cases typically require alternative treatments such as chemotherapy or targeted therapy [7,9].

Accurate assessment of PR status is essential for treatment planning. However, current manual evaluation of staining intensity and positive nuclei proportions is time-consuming and subject to inter-observer variability [10,11]. To address this, this study proposes an automated PR-IHC framework consisting of nuclei segmentation, classification into four intensity-based categories (negative, weak, moderate, strong), and scoring. This unified pipeline provides reproducible, efficient, and clinically interpretable results.

**Figure 1:**
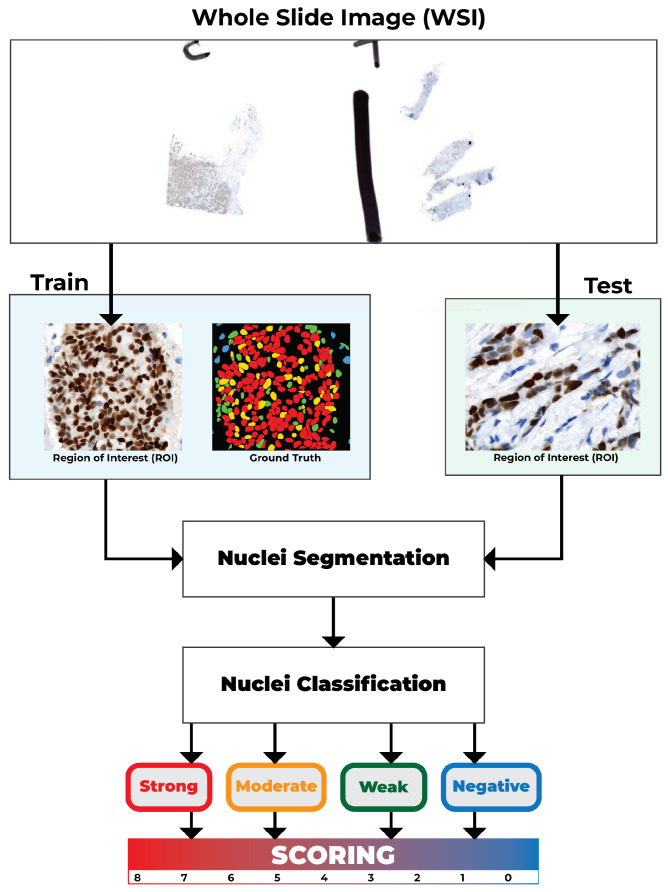
The overview of the proposed PR-IHC scoring system. The pipeline incorporates nuclei segmentation, classification into four intensity-based categories, and ROI-level scoring.

The block diagram of the proposed pipeline is shown in Figure 1, which shows nuclei segmentation using the Cellpose framework [12,13] and on DAB-intensity-based classification using automated scoring as separate modules [5,7,10]. This paper integrates these components into a single, end-to-end framework and evaluates their performance on a consistent dataset to objectively assess the system’s overall accuracy and reliability.

The nuclei segmentation stage addresses challenges present in PR-IHC slides, such as overlapping structures, unclear nuclear boundaries, and varied morphology [10]. The Cellpose model is employed to precisely delineate individual nuclei, providing dependable input for subsequent analysis [12]. Following segmentation, each identified nucleus is subjected to stain deconvolution, isolating the DAB channel for the calculation of intensity features [8]. Nuclei are split into four classes: negative, weak, moderate, and strong, determined by calibrated intensity thresholds. Negative nuclei are readily identifiable owing to their bluish hematoxylin staining; however, differentiating between weak, moderate, and strong positive nuclei is challenging due to the subtle variations in brown DAB intensity [10]. Our classification framework utilizes calibrated thresholds, which are validated through quantitative assessment against expert-annotated ground truth to address this issue.

The classified nuclei are subsequently directed to the final stage of the system, where the scoring procedure is executed [5]. The Allred score at the ROI level is derived from the combination of the proportion of positive nuclei and the dominant staining intensity, and this score is then compared with manual scoring by pathologists for validation. This study highlights the Allred system for hormone receptor quantification due to its extensive use in clinical reporting and its compatibility with established ground truth annotations, despite the relevance of the H score [11]. This represents one of the few integrated automated pipelines for PR-IHC analysis, which integrates nuclei segmentation, classification, and Allred scoring. The proposed system has the potential to enhance the reproducibility and efficiency of PR evaluation while reducing the manual workload for pathologists.

Recent studies have explored multi-task and end-to-end deep learning models for histopathology analysis; however, most existing approaches primarily focus on ER-IHC or patch-level predictions and lack nucleus-level interpretability. In contrast, this study focuses on PR-IHC and introduces a pipeline that integrates segmentation, interpretable classification, and scoring in a unified framework. Studies have also explored automated IHC analysis using publicly available datasets and explainable deep learning frameworks, particularly for HER2 biomarker assessment [14,15,16]. Although these works focus on HER2, they share similar staining characteristics and computational pipelines with PR-IHC, making them relevant for methodological comparison.

This work extends our previous conference studies on breast cancer histopathology analysis. In our earlier work, we explored nuclei segmentation in ER-IHC images using deep learning techniques [17], followed by automated nuclei segmentation in PR-IHC images using the Cellpose model [18]. Furthermore, we investigated real-time nuclei classification and Allred scoring for PR-IHC images [19]. Building upon these studies, the current work presents a unified and enhanced framework that improves segmentation robustness and integrates segmentation, classification, and scoring into a single pipeline.

The structure of this paper is outlined as follows. Section 2 describes all the recent studies related to this experiment, and Section 3 delineates the distinct stages of the proposed automated PR-IHC scoring pipeline and how we implemented this research experiment. The experimental results are outlined in Section 4, including precision, recall, F1, and IoU scores. Section 5 briefly describes the experiment process and model strength. Section 6 concludes the paper and presents potential directions for future research.

## 2. Related Work

In our prior work, we investigated deep learning-based nuclei segmentation for ER-IHC images using hybrid architectures [17]. Subsequently, a dedicated PR-IHC segmentation framework based on the Cellpose model was introduced, demonstrating strong performance across diverse nuclear morphologies [18]. Additionally, a real-time framework for nuclei classification and Allred scoring was developed to support automated biomarker quantification [19]. However, these studies were conducted independently and lacked a unified framework. The present study addresses this gap by integrating and extending these approaches into a more robust and generalized segmentation pipeline.

Automated immunohistochemical (IHC) scoring for breast cancer has moved on from traditional feature-based approaches to advanced deep learning systems that are capable of performing nuclei segmentation, per-nucleus intensity grading, and whole-slide scoring. Ahmad Fauzi et al. [5] created whole-slide ER-IHC scoring systems based on the Allred framework, using a three-stage workflow at the WSI level. They demonstrated significant agreement with pathologists concerning Allred scores. The study illustrated the necessity of precise nuclei detection and intensity estimation for producing reliable slide-level results, especially when the number of positively stained nuclei is low. This study laid the groundwork for subsequent investigations focused on ER. Ahmad et al. [20] showed that deep learning models can accurately find nuclei and sort them by how strongly they are stained at the cellular level.

Classical computer vision techniques additionally contributed to early progress in automated immunohistochemistry scoring. Mouelhi et al. [10] presented an unsupervised pipeline that incorporates a modified Laplacian operator, improved watershed segmentation, and color-based stain separation to classify four intensity categories: negative, weak, moderate, and strong. Their method allowed for quick processing and was highly consistent with pathologists. However, it was not very reliable across different scanners and staining conditions because it depended on color features that were designed manually.

The increasing number of annotated datasets and enhancements in computational power have resulted in the preeminence of deep learning models in nuclei segmentation and intensity classification tasks. Mahanta et al. [9] introduced IHC-Net, a fully convolutional network that integrates semantic segmentation with an ensemble classifier for four-class classification. The system worked very well, but splitting the segmentation and classification modules made the costs of annotation higher and the work of engineers more difficult. Tile-based WSI scoring methods, as investigated by Ahmad et al. [21], revealed that high-capacity CNNs, like DenseNet, can achieve competitive concordance with pathologists concerning ER Allred scoring and treatment recommendations. These models work at the layer level, so there are no outputs for each nucleus. This restriction makes it harder to explain things and makes it impossible to directly measure the proportions of positive nuclei, which is an important part of Allred scoring.

Recent research has focused on the integration of nuclei segmentation and intensity grading into a cohesive end-to-end architecture to improve efficiency and clinical relevance. Lin et al. [22] introduced ISCAS-Net, an encoder–decoder model trained without manual segmentation masks, utilizing a multi-stage learning strategy. Their method enhanced overall compatibility with pathologists and significantly reduced inference time, showing that integrated training can improve accuracy and make it easier to analyze whole slides.

Additionally, there have been significant advances in automating ER, but there are not many PR-specific IHC pipelines yet, even though PR uses the same Allred scoring system and is clinically linked to ER. Hasan et al. [23] showed that it is possible to quickly classify PR nuclei and scoring, showing that lightweight and deployable solutions are possible. To fill this gap, our research focuses on PR by combining data-efficient nuclei segmentation and classification in a single end-to-end pipeline. Compared to standard three-stage systems that focus on ER, our method puts more emphasis on compact models, calibrated and understandable decision rules, predictable runtime, and complete nucleus-level auditability, all while making sure that the scoring performance is strong.

## 3. Materials and Methods

This section provides a detailed description of the three stages preceding scoring: nuclei segmentation, nuclei classification, and intensity-based scoring. The Allred methodology for evaluating progesterone receptor (PR) status is subsequently detailed.

The proposed methodology builds upon our previously developed segmentation frameworks for ER-IHC and PR-IHC datasets [17,18]. Unlike prior works that focused on individual staining types or standalone models, the current approach introduces a hybrid and generalized pipeline that enhances segmentation accuracy and robustness across varying tissue characteristics.

### 3.1. Dataset

The dataset utilized in this study, designated PR-IHC-40x, was collaboratively created by Multimedia University (MMU) and the University Malaya Medical Centre (UMMC) to facilitate automated analysis of progesterone receptor immunohistochemistry (PR-IHC) in breast cancer. A total of fifty PR-stained invasive breast carcinoma glass slides from UMMC were digitized into whole-slide images (WSIs) using a 3DHistech Pannoramic DESK scanner (3DHISTECH Ltd., Budapest, Hungary) at 40× magnification.

Expert pathologists used the Cytomine collaborative annotation platform to mark regions of interest (ROIs). This platform allowed for region marking, quality control, and multi-user verification [24]. The StarDist instance segmentation model automatically created the first nuclei masks, which were then improved manually in Cytomine. The corrected binary masks were used as the “ground truth” for separating the nuclei. Senior pathologists manually reviewed and validated about 32,000 nuclei in 250 ROI images to make sure that the morphology and diagnosis were correct.

A four-class labeling scheme based on DAB intensity was used to classify PR expression. The nuclear categories were Strong, Moderate, Weak, and Negative. Pathologists oversaw the calibration of intensity thresholds, and the classification masks were improved over time to match clinical interpretation.

### 3.2. Nuclei  Segmentation Method

Accurate nuclei segmentation is essential for reliable biomarker quantification in PR-IHC images. In this study, the Cellpose model [12] was used for instance segmentation, leveraging both nuclear probability and flow-based representations. Unlike traditional semantic segmentation methods, this approach enables effective separation of overlapping nuclei, which is critical in densely packed and heterogeneous PR-IHC tissue regions.

The backbone network is structured as an encoder–decoder, comprising eight residual convolutional blocks. The encoder comprises four downsampling stages, each consisting of two convolutional layers with 3 × 3 kernels, batch normalization, and rectified linear unit (ReLU) activation, succeeded by 2 × 2 max pooling for spatial reduction. The decoder replicates this architecture through four upsampling stages that integrate bilinear upsampling with convolutional layers and skip connections from the encoder, thereby maintaining fine-grained spatial details. Residual connections are utilized to enhance gradient flow stability and facilitate convergence. The final output layer comprises two branches: a probability map that predicts nuclear versus background pixels and a two-channel flow map that encodes horizontal and vertical displacement vectors.

The dataset consisted of 250 PR-IHC images obtained at 40× magnification, each paired with validated binary ground truth masks. The dataset was partitioned into 80% for training (200 images) and 20% for testing (50 images). Data augmentation techniques such as horizontal flipping, rotation, and intensity normalization were implemented to enhance variability in training. Model optimization utilized the Adam optimizer, commencing with an initial learning rate of $1\times 10^{-4}$. The training objective integrated cross-entropy loss for probability maps with mean squared error loss for flow vectors.

During inference, each PR-IHC region of interest (ROI) is analyzed using the trained Cellpose model to generate probability and flow maps. A probability threshold is utilized to eliminate background regions, followed by clustering of flow vectors to reconstruct distinct nuclear instances. Postprocessing involves the elimination of spurious small objects (area < 20 pixels) and the consolidation of fragmented masks. The final output is a binary mask that delineates individual nuclei.

Algorithm 1 summarizes the entire training and inference pipeline of the fine-tuned Cellpose model, which has been used for segmenting nuclei in PR-IHC images. The model was initialized with pretrained Cellpose weights and optimized by a supervised learning process jointly minimizing probability and flow-map reconstruction losses. In every iteration of training, the model takes in augmented image–mask pairs and passes them through the residual U-Net backbone to learn both nuclear probability maps and pixel-wise displacement vectors. Model selection and saving of checkpoints were guided by various validation metrics including Dice and IoU. At test time, a trained model predicts probability and flow that are converted into instance-level masks via the flow integration mechanism of Cellpose, followed by size filtering and morphological postprocessing. This allows for robust segmentation of densely packed nuclei with irregular shapes in IHC slides by incorporating flow-based instance reconstruction and probability thresholding.
**Algorithm 1** Fine-tuning and inference procedure for Cellpose-based nuclei segmentation**Require:** 
Training set $D_{\mathrm{train}}$, validation set $D_{\mathrm{val}}$, pretrained weights $\Theta_{\mathrm{pre}}$, batch size *B*, learning rate η, epochs *N***Ensure:** 
Fine-tuned weights $\Theta^{*}$ and inference function InferCellpose  1:**Initialize:** Load Cellpose architecture and $\Theta \leftarrow \Theta_{\mathrm{pre}}$  2:Set optimizer opt←Adam(Θ;η) and loss weights $\lambda_{\mathrm{prob}},\lambda_{\mathrm{flow}}$  3:**for** epoch=1 to *N* **do**  4:      **for** each minibatch $\left(X_{b},Y_{b}\right)$ from $D_{\mathrm{train}}$ **do**  5:            Augment $\left(X_{b},Y_{b}\right)$  6:            Predict $P,F\leftarrow {\mathrm{Model}}_{\Theta }\left(X_{b}\right)$  7:            $L_{\mathrm{prob}}\leftarrow \mathrm{CrossEntropy}\left(P,Y_{\mathrm{prob}}\right)$  8:            $L_{\mathrm{flow}}\leftarrow \mathrm{MSE}\left(F,Y_{\mathrm{flow}}\right)$  9:            $L\leftarrow \lambda_{\mathrm{prob}}L_{\mathrm{prob}}+\lambda_{\mathrm{flow}}L_{\mathrm{flow}}$10:            Backpropagate and opt.step(), opt.zero_grad()11:      **end for**12:      Evaluate on $D_{\mathrm{val}}$, save checkpoint if improved13:**end for**14:**function** InferCellpose($X,\Theta_{\mathrm{best}},\tau_{\mathrm{prob}},A_{\min}$)15:      $P,F\leftarrow {\mathrm{Model}}_{\Theta_{\mathrm{best}}}\left(X\right)$16:      $M_{\mathrm{bin}}\leftarrow \left(P\geq \tau_{\mathrm{prob}}\right)$17:      Reconstruct instances from *F* inside $M_{\mathrm{bin}}$ (flow integration)18:      Postprocess: remove area $<A_{\min}$, fill holes, merge fragments19:      **return** $M_{\mathrm{inst}}$20:**end function**

**Figure 2:**
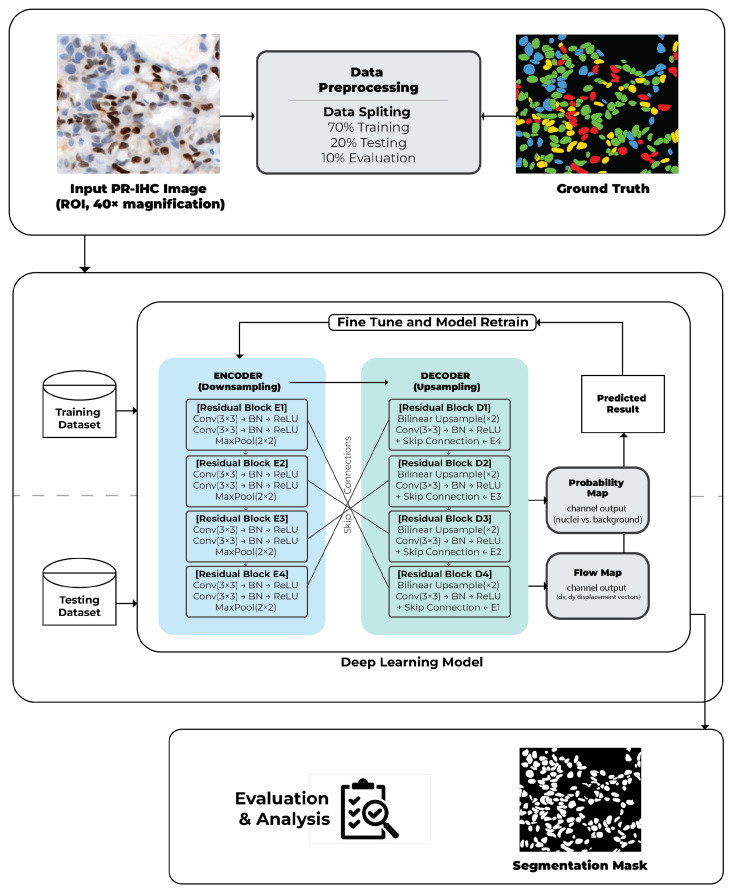
The original PR-IHC input image is processed by the Cellpose model, which predicts probability and flow maps that are combined into instance-level binary masks representing individual nuclei.

Figure 2 illustrates the overall segmentation workflow, showing an example PR-IHC input image and the corresponding binary mask produced by the model.

All experiments were implemented in Python (version 3.9, Python Software Foundation, Wilmington, DE, USA) using Jupyter Notebook (version 6.4, Project Jupyter). The model was trained for 250 epochs using the Adam optimizer (implemented in PyTorch, version 1.12, Meta AI, Menlo Park, CA, USA) with a learning rate of $1\times 10^{-4}$. Data augmentation techniques included rotation, flipping, and intensity normalization. Experiments were conducted on a system equipped with an Intel Core i7-1065G7 CPU (Intel Corporation, Santa Clara, CA, USA) and 16 GB RAM.

#### 3.2.1. Experiment for Comparison with Additional Deep Learning Segmentation Models

The proposed segmentation framework was evaluated for its applicability to PR-IHC image analysis by comparing the fine-tuned Cellpose model with various established deep learning architectures. Three supplementary models have been incorporated for methodological comparison: the baseline Cellpose model, U-Net, and StarDist. The chosen models exemplify various segmentation methodologies and function as standard benchmarks in the field of biomedical image analysis.

The baseline Cellpose model, with pretrained standard microscopy weights, was used as a benchmark to assess enhancements achieved via domain-specific fine-tuning. The baseline model, although successful across several imaging modalities, is not tailored for the distinct chromogenic profiles and tightly clustered nuclei commonly observed in PR-IHC tissue slices. This comparison enabled the assessment of the advantages gained from customizing the architecture to the specific staining and morphological traits of the dataset.

The U-Net architecture was selected as a representative example of classical encoder–decoder segmentation networks. U-Net utilizes a contracting path to acquire contextual information and an expansive path that reinstates spatial resolution through the use of skip connections. U-Net is effective for semantic segmentation; however, it lacks inherent support for instance separation. Consequently, further postprocessing steps, including watershed segmentation, are necessary to derive individual nuclear masks. This feature establishes U-Net as a critical benchmark for assessing the performance enhancements of specialized instance-segmentation architectures in densely packed PR-IHC regions.

StarDist serves as an example of a shape-based instance segmentation method. StarDist models each nucleus as a star-convex polygon, characterized by radial distances from its center, facilitating the effective differentiation of objects with nearly circular shapes. This design is notably effective for fluorescence microscopy, as nuclei generally display uniform shapes and intensity distributions. The inclusion of StarDist in the comparison illustrates the interaction between shape priors and the irregular nuclear boundaries, as well as the variable staining patterns characteristic of PR-IHC slides.

These models establish a diverse methodological baseline for contextualizing the proposed fine-tuned Cellpose approach. This study compares architectures utilizing semantic segmentation (U-Net), polygon-based reconstruction (StarDist), and generic instance segmentation weights (baseline Cellpose) to establish a framework for selecting the optimal segmentation strategy for PR-IHC biomarker quantification.

#### 3.2.2. Cross-Dataset Evaluation Strategy

An additional experiment was designed to evaluate the generalization capability of the segmentation pipeline by applying the trained model to a secondary immunohistochemical marker. An external ER-IHC dataset was prepared using the same preprocessing and annotation pipeline as that employed for PR-IHC images. This evaluation was designed to assess the applicability of a model trained on PR-stained tissue to images exhibiting varying staining characteristics, rather than to report performance outcomes.

In this cross-dataset experiment, the fine-tuned Cellpose model, which was trained solely on PR-IHC images, was applied directly to ER-IHC regions of interest without further training or parameter modifications. This configuration enabled the evaluation of the segmentation workflow amidst the staining, chromogen, and morphological variability characteristic of actual clinical data. The identical postprocessing procedures, comprising probability thresholding, flow-based instance reconstruction, and small object removal, were preserved to ensure methodological consistency across datasets.

The addition of the segmentation pipeline offers a methodological framework for assessing robustness and transferability, essential factors in the development of AI-assisted biomarker quantification tools designed for multimarker clinical workflows.

### 3.3. Nuclei  Classification Method

After segmentation, each identified nucleus is categorized into one of four expression levels: Strong, Moderate, Weak, or Negative. This task is conducted according to the intensity of the 3,3′-diaminobenzidine (DAB) chromogen, which acts as an indicator of progesterone receptor expression in PR-IHC slides.

DAB serves as a brown chromogenic substrate in immunohistochemistry, precipitating at the site of antigen expression upon binding with horseradish peroxidase–linked antibodies [25]. Nuclei exhibiting elevated receptor expression are characterized by a darker brown coloration, whereas unstained or negative nuclei maintain the bluish tint of the hematoxylin counterstain. Stain deconvolution was utilized to isolate the hematoxylin and DAB channels. The DAB channel produced a grayscale representation of biomarker expression appropriate for computational analysis.

Darker nuclei indicate stronger expression; therefore, the DAB intensity was inverted to associate higher expression values with lower pixel intensities. In this representation, negative nuclei with a bluish counterstain exhibit values approaching 1.0, whereas strongly positive nuclei with dark brown staining present values around 0.80 or lower. This inversion guarantees that classification aligns with pathologists’ interpretations, wherein darker staining indicates greater receptor expression.

**Figure 3:**
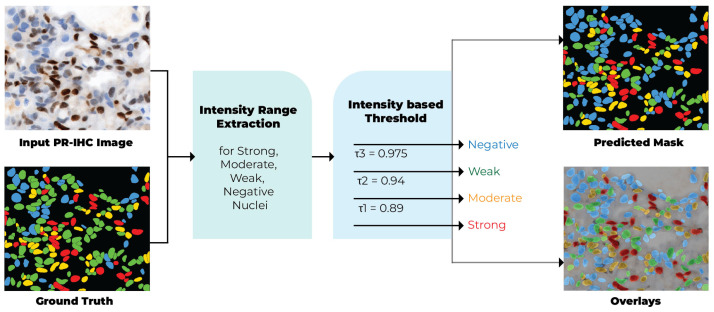
Process of the nuclei classification phase. The initial PR-IHC input image undergoes stain deconvolution and intensity thresholding, yielding four-color masks that denote Negative (blue), Weak (green), Moderate (yellow), and Strong (red) nuclei.

As illustrated in Figure 3, the overall nuclei classification workflow begins with the PR-IHC input image, followed by stain deconvolution and intensity thresholding, ultimately producing four-color masks representing Negative (blue), Weak (green), Moderate (yellow), and Strong (red) nuclei.

For each segmented nucleus region $\Omega_{i}$, the mean DAB intensity was calculated as:

(1) $$\mu_{DAB}^{i}=1-\frac{1}{|\Omega_{i}|}\sum_{\left(x,y\right)\in \Omega_{i}}DAB\left(x,y\right),$$

where $|\Omega_{i}|$ represents the number of pixels in nucleus *i*, and DAB(x,y) is the DAB channel value at pixel coordinates (x,y). The subtraction from 1 inverts the values such that lower intensities correspond to stronger staining.

Classification was performed by applying three calibrated thresholds $\left(\tau_{1},\tau_{2},\tau_{3}\right)$ to divide the nuclei into four categories:

(2) $$C_{i}=\left\{\begin{array}{cc} \mathrm{Strong}, & \mu_{DAB}^{i}<\tau_{1},\\ \mathrm{Moderate}, & \tau_{1}\leq \mu_{DAB}^{i}<\tau_{2},\\ \mathrm{Weak}, & \tau_{2}\leq \mu_{DAB}^{i}<\tau_{3},\\ \mathrm{Negative}, & \mu_{DAB}^{i}\geq \tau_{3}. \end{array}\right.$$

To identify the threshold range, we used four ROI images and Ground truth to extract intensity and generate the threshold range. After calibrating, we found the threshold values of $\tau_{1}=0.89$, $\tau_{2}=0.94$, and $\tau_{3}=0.975$. Upon establishment, the thresholds were fixed and directly applied to all 250 ROIs. To evaluate performance, we used 20% of the Images. We extracted one image from one WSI, so in total we used 50 ROIs from 50 WSIs to ensure all types of data were tested.

Threshold calibration was performed using a small set of representative ROIs to enable rapid and lightweight adaptation. This design allows the system to operate with minimal ground truth samples while maintaining robustness. The calibrated thresholds were subsequently applied to all 250 ROIs (32,000 nuclei), ensuring large-scale evaluation and reducing the risk of overfitting.

Additionally, the method was validated on an independent ER-IHC dataset without recalibration, demonstrating generalizability across biomarkers.

This intensity-based approach provides a lightweight and robust method for nuclei classification, directly reflecting staining intensity variations observed in clinical practice. It ensures reproducible results across different images while maintaining interpretability and efficiency.

**Figure 4:**
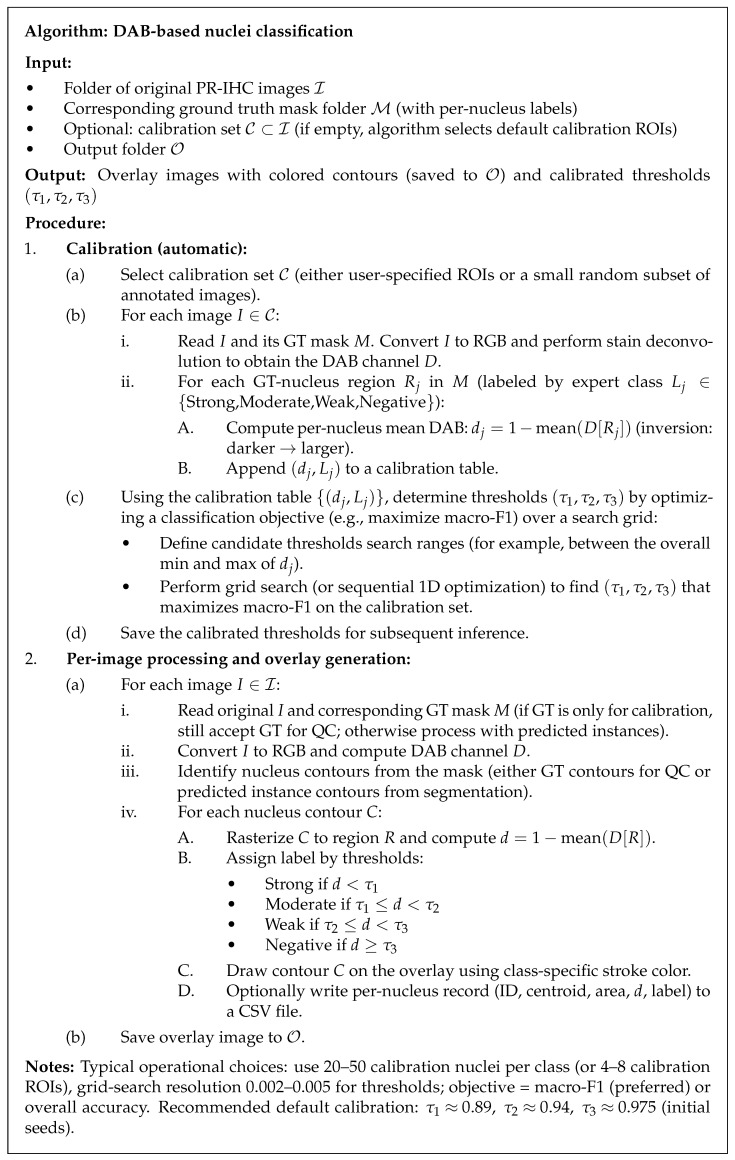
DAB-based nucleus classification with automatic threshold calibration.

The algorithm in Figure 4 extends the deterministic DAB-intensity classification workflow with an automatic calibration step to determine the threshold values $\left(\tau_{1},\tau_{2},\tau_{3}\right)$ from expert-annotated ground truth. During calibration, mean DAB values per nucleus are computed in a small and representative set of annotated ROIs and paired with their GT class labels to form a calibration table. Optimal thresholds are then determined by a simple optimization (grid search) that maximizes an adopted objective of interest on the calibration set, such as macro-F1, thus optimizing for the staining characteristics and scanner profile of the study cohort. These thresholds are fixed once computed and, in the bulk image inference stage, are used deterministically to decide on Strong/Moderate/Weak/Negative labels per nucleus; the same deep-learning classifiers previously described may be used in parallel for comparison or hybrid decision schemes. This automated calibration step aims to enhance reproducibility by minimizing manual adjustment of thresholds while preserving the interpretability and auditability of the DAB-intensity method. See methodology and dataset details.

#### Extended Classification Framework and Cross-Marker Evaluation

In addition to the intensity-based classification strategy, the methodology incorporates deep learning architectures to capture morphological and textural features beyond intensity. Image patches centered on each nucleus were extracted and standardized through resizing, normalization, and augmentation. Three models were used: a lightweight CNN, ResNet-18, and EfficientNet-B0. The CNN served as a basic feature extractor, ResNet-18 improved stability through residual connections, and EfficientNet-B0 provided efficient scaling. Each model was adapted for four-class classification and trained using cross-entropy loss. This setup complements the intensity-based method by providing a flexible framework for learned feature representation.

To assess the versatility of this classification framework across various immunohistochemical markers, the identical methodological pipeline was implemented on an estrogen receptor (ER) IHC dataset. The preprocessing steps for the ER images were the same as those for the PR-IHC classification. These steps included stain deconvolution, nuclear extraction, and patch preparation. Significantly, no further fine-tuning or threshold recalibration was conducted; both the DAB-intensity classifier and the deep learning models, which were trained solely on PR-IHC nuclei, were directly applied to ER-IHC patches. This experimental design enabled the assessment of the framework amidst natural fluctuations in chromogen density, staining protocols, and tissue morphology. Keeping a consistent workflow across markers lets you check the robustness of cross-dataset comparisons and shows that the pipeline works for a wide range of methods without making changes or biases that are specific to any one marker.

### 3.4. Scoring Method

**Figure 5:**
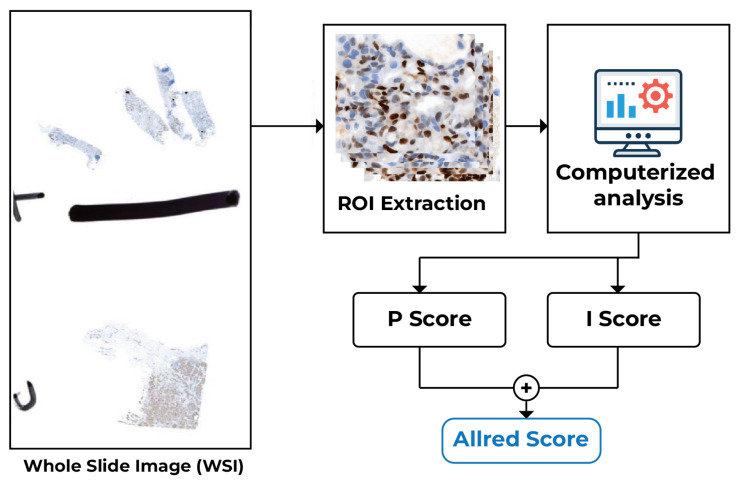
Automated scoring workflow for whole-slide images from PR-IHC. The final Allred score (P+I) is found by taking representative ROIs, breaking them up into nuclei, and putting them into four intensity levels.

This study analyzed 50 whole-slide images (WSIs) of progesterone receptor (PR) immunohistochemistry (IHC) to assess hormone receptor expression using the scoring method. From each WSI, representative regions of interest (ROIs) were extracted to concentrate on diagnostically significant tumor regions. We used computers to analyze these ROIs, which included segmenting the nuclei and classifying them into four groups based on intensity: Negative, Weak, Moderate, and Strong. Using the classification results, the proportion score (*P*) and intensity score (*I*) were calculated to get the final Allred score (P+I). This gave a numerical measure of PR expression at the ROI and WSI levels. Figure 5 shows how the whole process of this computational scoring system works.

The scoring system to assess progesterone receptor (PR) expression. The Allred score integrates two complementary metrics: the proportion score (*P*), indicating the percentage of PR-positive nuclei, and the intensity score (*I*), which denotes the staining strength of the predominant positive nuclei. The final Allred score is the aggregate of these two values, with a range from 0 to 8. Scores of 0 and 2 are classified as negative (not actionable), whereas scores ranging from 3 to 8 are deemed positive, suggesting that hormonal therapy is recommended for the patient.

Formally, the Allred score is given by:

(3) AllredScore=P+I

where *P* is the proportion score and *I* is the intensity score.

Table 1 and Table 2 summarize the scoring criteria for *P* and *I*, respectively.

The final Allred score is computed by summing the two components:

The relationship between the proportion score (P), intensity score (I), and the final Allred score is summarized in Table 3, which illustrates how different combinations of P and I result in an overall score ranging from 0 to 8.

**Figure 6:**
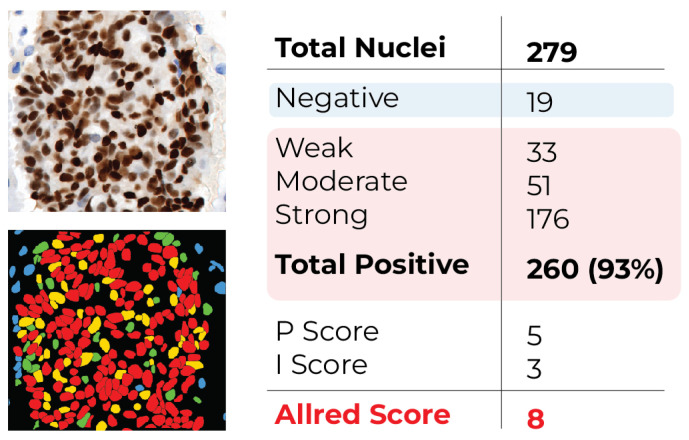
Illustration of scoring derived from experimental results (Image ID: 318816960). Left: Original input image for PR-IHC. Classified output mask displaying nuclei color-coded as Negative (blue), Weak (green), Moderate (yellow), and Strong (red). The computed Allred score for this image was 8, with P = 5 and I = 3.

The scoring process is illustrated by Figure 6. The segmentation and classification pipeline identified a total of 279 nuclei. A total of 176 were categorized as Strong, 51 as Moderate, 33 as Weak, and 19 as Negative. The proportion of positive nuclei (Strong + Moderate + Weak) was thus calculated as follows:

(4) $$\mathrm{Positive}\;\mathrm{Percentage}=\frac{176+51+33}{279}\times 100=93.18\%$$

Table 1 and Table 2 indicate a proportion score of P=5. And the intensity score was I=3, as the majority of positive nuclei (176 nuclei) exhibited strong staining. Consequently, the final Allred score is:

(5) AllredScore=P+I=5+3=8

This example illustrates that the automated pipeline accurately assigns a high Allred score to PR-IHC images characterized by pronounced nuclear staining, aligning with the pathologists’ ground truth assessment. Figure 6 illustrates this case, presenting the original PR-IHC input image in conjunction with the classified output mask. Nuclei in the mask are color-coded as Negative (blue), Weak (green), Moderate (yellow), and Strong (red).

### 3.5. Evaluation Metrics

To evaluate the performance of the segmentation models, standard metrics including precision, recall, F1-score, and intersection over union (IoU) are employed. These metrics are widely used in deep learning-based medical image analysis to ensure robust and reliable assessment of classification and segmentation performance across various biomedical applications [26,27].

Precision measures the proportion of correctly predicted positive pixels among all predicted positives:

(6) $$\mathrm{Precision}=\frac{TP}{TP+FP}$$

Recall (Sensitivity) measures the proportion of correctly predicted positive pixels among all actual positives:

(7) $$\mathrm{Recall}=\frac{TP}{TP+FN}$$

F1-score is the harmonic mean of Precision and Recall, providing a balanced measure of segmentation performance:

(8) $$\text{F1-score}=2\times \frac{\mathrm{Precision}\times \mathrm{Recall}}{\mathrm{Precision}+\mathrm{Recall}}$$

Intersection over Union (IoU) evaluates the overlap between predicted and ground truth segmentation masks:

(9) $$\mathrm{IoU}=\frac{TP}{TP+FP+FN}$$

where TP (True Positive) represents correctly segmented nuclei pixels, FP (False Positive) represents incorrectly predicted nuclei pixels, and FN (False Negative) represents missed nuclei pixels.

For nucleus-level classification, macro-F1 was used to ensure balanced evaluation across all classes (Strong, Moderate, Weak, and Negative), regardless of class imbalance.

(10) $$\text{Macro-F1}=\frac{1}{C}\sum_{i=1}^{C}F1_{i}$$

where *C* is the total number of classes and $F1_{i}$ represents the F1-score of class *i*.

Precision reflects how accurately the model avoids false detections, while Recall indicates its ability to detect all relevant nuclei. The F1-score balances both metrics, making it suitable for imbalanced datasets. IoU provides a strict measure of segmentation quality by quantifying spatial overlap. Macro-F1 ensures that all classes contribute equally to the final classification performance, which is particularly important in multi-class histopathological analysis.

## 4. Results

The outcomes of the proposed PR-IHC analysis pipeline are delineated in three sections. Initially, we assess the nuclei segmentation stage, which serves as the basis for subsequent biomarker quantification. The performance of the nuclei classification module is presented, which categorizes each segmented nucleus into one of four staining categories according to DAB intensity [8]. We evaluate the comprehensive scoring method, integrating proportion and intensity scores to produce clinically interpretable outcomes [5]. The subsequent subsections provide a detailed description of each stage, incorporating both quantitative and qualitative evaluations [6].

### 4.1. Nuclei Segmentation

**Figure 7:**
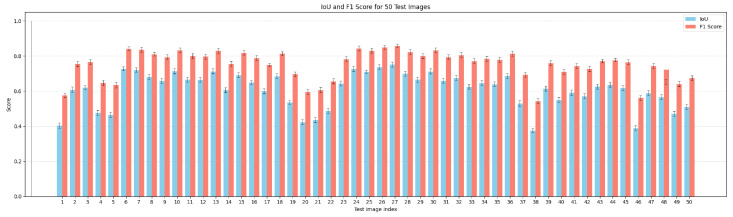
Bar chart of segmentation performance across 50 PR-IHC test images, showing F1 score, and IoU for each image.

Both quantitative and qualitative evaluations were performed to evaluate the performance of the nuclei segmentation stage on the 50 test images. The Cellpose-based segmentation framework exhibited consistent instance-level delineation in diverse PR-IHC tissue samples. Figure 7 illustrates a bar chart that summarizes performance metrics for the 50 test images. The system attained an average Precision of 0.8882, Recall of 0.8215, F1 score of 0.8535, and Intersection over Union (IoU) of 0.7445.

**Figure 8:**
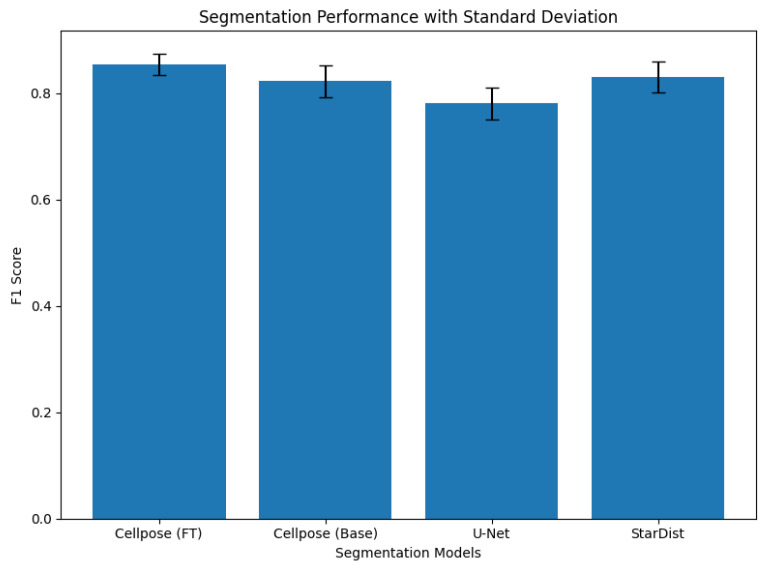
Comparison of segmentation performance across different models using F1-score. Error bars represent the standard deviation across 50 test images, indicating the stability and robustness of each model.

Table 4 and Figure 8 demonstrate the results for the four models we used in this experiment. The Cellpose baseline, U-Net, and StarDist models all showed much lower segmentation performance across all evaluation metrics than the fine-tuned Cellpose model. The Cellpose baseline was somewhat accurate, but it could not find the exact edges of the boundaries, especially in areas with clustered nuclei. U-Net got the lowest scores overall because it had trouble separating nuclei that were touching or overlapping, which often led to merged segmentations. StarDist did better than U-Net and got close to the baseline Cellpose performance. This was because it used a star-convex polygon representation. However, it still had trouble with nuclei that were not shaped like stars and regions that were not stained well. These comparative results underscore the need to optimize Cellpose to adeptly manage the morphological variability found in PR-IHC tissue samples.

The segmentation results demonstrate high detection accuracy, with strong Precision and Recall indicating reliable identification of nuclei. The F1-score reflects consistent performance across the dataset, while the IoU confirms good spatial agreement with ground truth masks. These results establish a strong foundation for subsequent classification and scoring stages.

**Figure 9:**
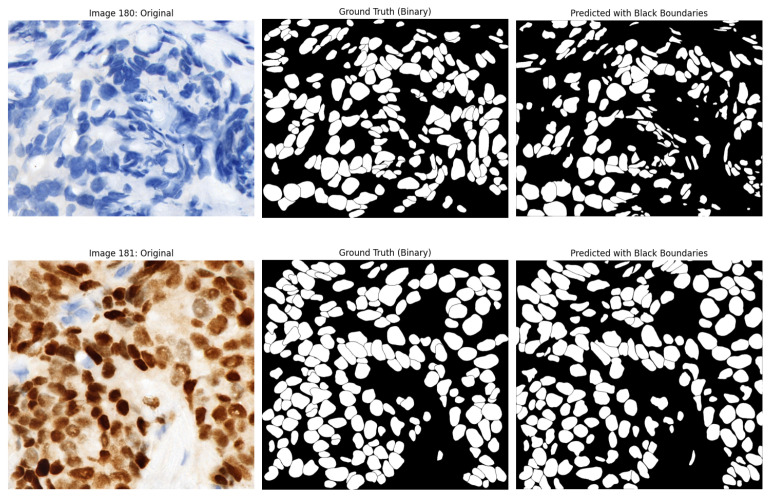
Representative segmentation results on PR-IHC test images. (**Left**): Original PR-IHC images. (**Middle**): Ground truth annotations. (**Right**): Predicted segmentation masks.

The qualitative analysis elucidates the strengths and limitations of the segmentation model. Figure 9 presents representative examples. Nuclei in well-stained images were segmented with precision, exhibiting clear boundary preservation and a low incidence of false positives. Conversely, regions with weak staining or dense clustering exhibited under-segmentation and sporadic boundary inconsistencies, resulting in the omission or merging of nuclei. Overall segmentation performance remained stable despite the challenges presented by varying staining conditions.

The findings indicate that the Cellpose-based segmentation [12] stage establishes a robust basis for subsequent classification and scoring. The system facilitates precise downstream biomarker quantification by consistently isolating individual nuclei, even under difficult PR-IHC conditions.

#### Segmentation Performance on ER-IHC Dataset

An independent evaluation of the 44 ER-IHC test images was carried out to ensure the robustness of the segmentation architecture. The dataset revealed greater variability in staining and reduced chromogenic contrast compared to PR-IHC samples, challenging data differentiation. However, the model demonstrated a Precision of 0.7200, a Recall of 0.8420, an F1 Score of 0.7468, and an IoU of 0.6018, indicating its efficacy across various biomarker staining profiles.

Quantitative segmentation performance on the ER-IHC dataset is reported in Table 5, presenting Precision, Recall, F1-score, and IoU values across 44 ER-IHC test images.

**Figure 10:**
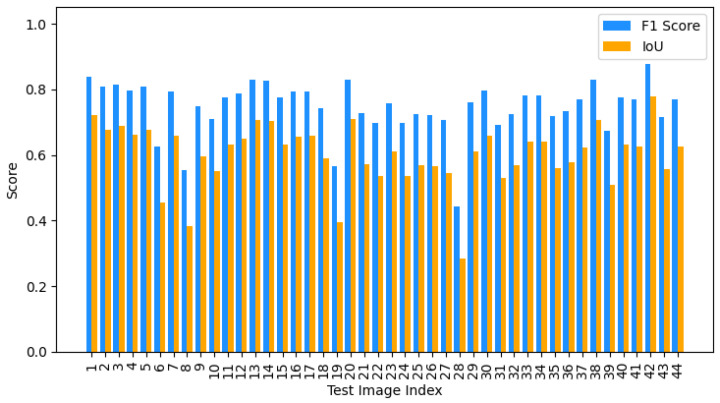
Bar chart showing average F1 Score and IoU for 44 ER-IHC test images.

Figure 10 illustrates the bar chart that encapsulates the average F1 score and IoU for the ER-IHC test images. Despite the segmentation accuracy being inferior to PR-IHC due to diminished nuclear contrast in ER-stained slides, the model exhibits consistent generalization across biomarkers, underscoring its flexibility in diverse IHC settings.

**Figure 11:**
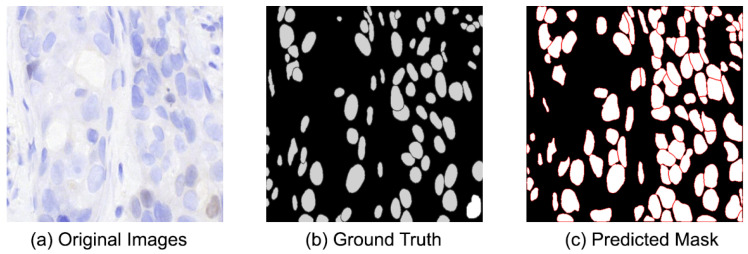
Representative segmentation results on ER-IHC test images. (**a**) Original ER-IHC image. (**b**) Ground truth annotations. (**c**) Predicted nuclei segmentation mask.

Alongside the quantitative results, qualitative examples illustrate the model’s performance on ER-IHC slides. Figure 11 displays ER-IHC original images with their corresponding ground truth and predictions. Despite the weaker and more varied nature of ER staining, the model typically succeeds in accurately detecting and characterizing the majority of nuclei. Under-segmentation persists, particularly in regions with densely clustered nuclei; however, the predictions are generally uniform across various staining conditions.

### 4.2. Nuclei Classification

The second stage of the pipeline entailed the classification of segmented nuclei into four categories based on staining intensity: Strong, Moderate, Weak, and Negative. The DAB intensity-based thresholding strategy outlined in Section 3.3 was employed for this task. The performance evaluation was carried out on 50 test images, utilizing pathologist-verified ground truth masks.

The classification performance is summarized in Table 6, detailing Precision, Recall and F1 score for each class. The proposed method demonstrated consistently high performance across all categories, achieving a macro-averaged Precision of 0.9482, Recall of 0.9494 and F1 score of 0.9487. The results indicate that the pipeline effectively differentiates nuclei based on varying staining intensities.

The reported values represent mean performance across the test dataset. The proposed DAB-intensity classifier exhibited low variability (F1-score ± 0.01), demonstrating robust and consistent classification performance across diverse nuclei staining patterns, while maintaining competitive accuracy compared to deep learning-based models.

**Figure 12:**
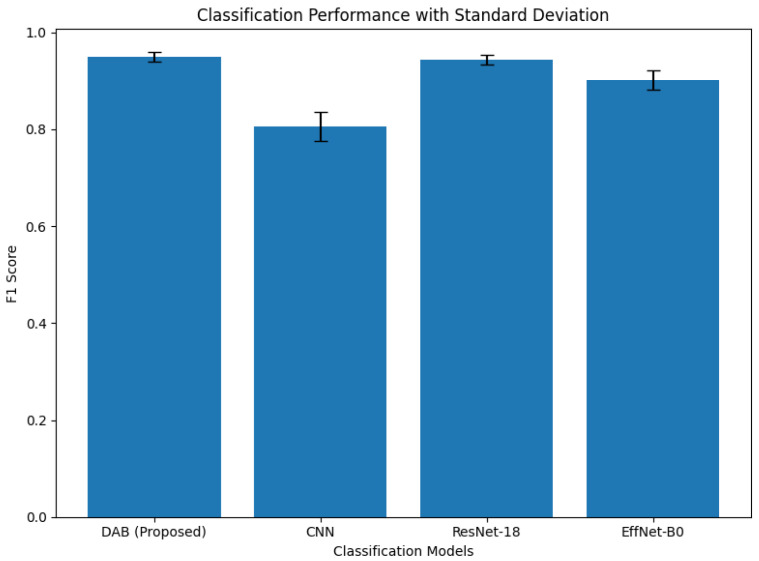
Comparison of classification performance across models using F1-score. Error bars represent the standard deviation across test samples, demonstrating the robustness of the proposed DAB-intensity classifier.

A comparative evaluation between the proposed DAB-intensity classifier and representative deep learning models is presented in Table 7 and Figure 12 highlighting differences in precision, recall, F1-score, and accuracy.

Compared to the proposed DAB-intensity classifier, deep learning models such as CNN, ResNet-18, and EfficientNet-B0 showed lower or less consistent performance. While ResNet-18 achieved competitive results, it required significantly higher computational resources and training time. In contrast, the proposed method operates efficiently without training, providing stable and interpretable classification suitable for real-time clinical applications.

Conversely, the DAB-Intensity classifier attained outstanding results across all measures while functioning in real time, without requiring model training or extensive datasets. The rule-based intensity measurement renders it highly interpretable and robust to staining variations, facilitating accurate and quick classification appropriate for clinical use.

**Figure 13:**
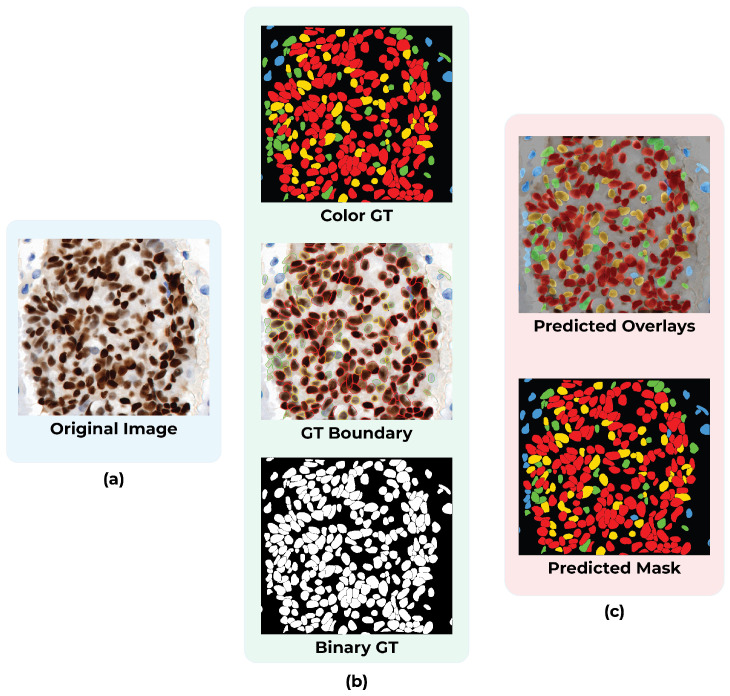
Representative classification results. (**a**): Original PR-IHC input images. (**b**): Ground truth annotations with four-class labels. (**c**): Predicted classification masks, where nuclei are color-coded as Negative (blue), Weak (green), Moderate (yellow), and Strong (red).

Figure 13 shows representative qualitative results. The figure presents a comparison of original PR-IHC images (left), ground truth annotations (middle), and predicted classification masks (right). The predicted masks demonstrate the model’s capacity to accurately classify nuclei into Negative (blue), Weak (green), Moderate (yellow), and Strong (red) categories, aligning closely with the ground truth.

**Figure 14:**
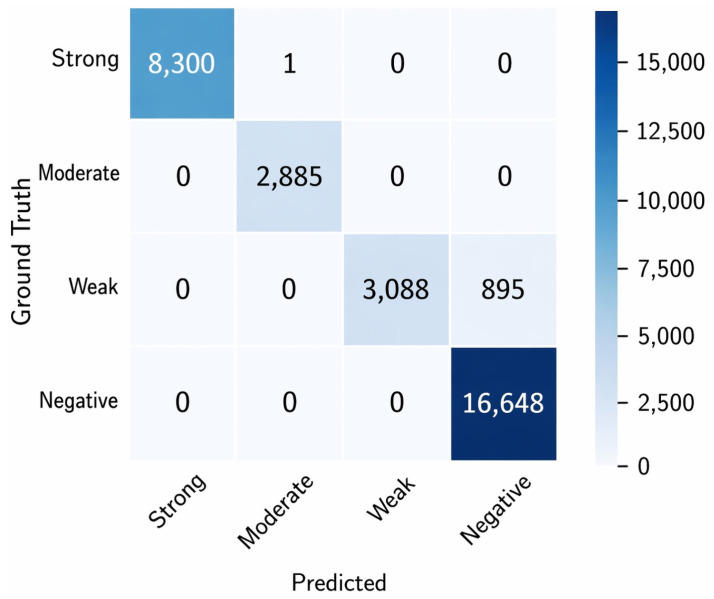
Confusion matrix for nucleus-level PR-IHC classification computed on ∼32,000 nuclei extracted from 250 ROIs derived from 50 PR-stained whole-slide images (WSIs) from UMMC.

A confusion matrix was constructed to analyze classification performance further (Figure 14). The findings reveal that most misclassifications transpired between the Weak and Moderate categories. The boundary between weak and moderate staining is often subtle, even for human experts, which makes this expectation reasonable. Strongly stained and negative nuclei were classified with high accuracy.

The DAB intensity-based classification stage demonstrated consistent and reliable performance across diverse PR-IHC slides. The elevated macro-averaged F1 score and IoU indicate that the system effectively captures nucleus-level variability with high fidelity. Borderline staining intensities created ambiguity between the Weak and Moderate classes; however, the classification accuracy for clinically significant categories (Strong and Negative) was excellent. The findings confirm the reliability of the classification stage as a basis for precise downstream scoring.

#### Classification Performance on ER-IHC Dataset

To enhance the generalization evaluation among biomarkers, the classification model was further applied to 44 test images of ER-IHC. The ER dataset is less stained by DAB compared to PR-IHC slides and contains greater variation, making classification challenging. Nevertheless, the performance of the model turned out to be quite good, with a Precision of 0.9380, a Recall of 0.9010, and an F1 Score of 0.9110, proving that the DAB-based classification technique remains robust even under challenging conditions of staining.

The classification performance of the proposed method on ER-IHC test images is summarized in Table 8, reporting Precision, Recall, and F1-score across the dataset.

**Figure 15:**
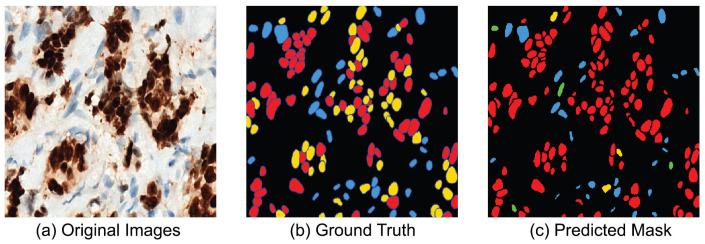
Representative classification results on ER-IHC images. (**a**) Original ER-IHC image. (**b**) Ground truth labels. (**c**) Predicted classification mask showing nuclei categorized as Negative (blue), Weak (green), Moderate (yellow), and Strong (red).

Figure 15 depicts a selection of non-quantitative examples from the ER-IHC dataset. The classifier appropriately assigns nuclei into their correct staining category, despite lower contrast and reduced intensity of staining. There is some confusion between the Weak and Moderate classes due to small tone differences. However, the overall accuracy of the classification stays the same across different ER staining patterns.

### 4.3. Scoring

The Allred scores for the 50 PR-IHC test images were calculated by aggregating classifications at the nucleus level. Each slide’s proportion of positive nuclei (Weak + Moderate + Strong) resulted in the proportion score P, while the dominant positive intensity provided the intensity score I; the score is calculated as P + I (range 0–8). Table 9 presents automated results for all 50 images.

The automated system achieved an exact match with the manual Allred score in 35 out of 50 cases (70.0%). It was within ±1 in 43 cases (86.0%) and within ±247 cases (94.0%). The concordance of treatment recommendations, defined as negative for scores 0 and 2 and positive for scores 3 to 8, was 47 out of 50, representing 94.0%. In a sample of slides with a manual score of 0 (n=12), the system accurately identified nine as correct zeros. The remaining three slides exhibited minor predicted positive fractions, resulting in two being scored as 3 and one as 2. Mitigation of these edge cases can be achieved through the implementation of a low-positive floor rule and tile-level background-brown quality control, ensuring that clearly positive slides remain unaffected.

## 5. Discussion

The proposed pipeline was validated through benchmarking against multiple segmentation and classification baselines, as well as cross-dataset evaluation on PR and ER-IHC images, demonstrating strong performance and reliable generalization across biomarkers. The fine-tuned Cellpose backbone adapts effectively to hormone-receptor IHC by learning stain-specific nuclear textures and boundary characteristics. Residual connections improve robustness to complex nuclear morphology and dense clustering, while the flow and probability branches enhance separation of overlapping nuclei and accurate foreground detection. These adaptations result in a segmentation model better suited to chromogenic IHC images than the standard Cellpose.

A comparison between the proposed PR-IHC scoring pipeline and existing computational pathology studies is provided in Table 10, outlining methodological contributions and reported performance across different biomarkers and datasets.

The proposed DAB-intensity classifier is a lightweight, threshold-calibrated, stain-aware method for per-nucleus classification. Unlike deep learning approaches, it does not require large annotated datasets or GPU resources, while still achieving competitive performance. Its deterministic formulation improves interpretability and robustness to staining variations, making it suitable for practical clinical deployment.

This research demonstrates that a practical, fully automated pipeline, starting with nuclei-level instance segmentation and advancing through stain-aware classification to slide-level scoring, can be reliably implemented for PR-IHC breast carcinoma [5,23]. The pipeline integrates a generalist, nuclei-aware segmentation backbone with DAB-channel quantification, reflecting the pathologist’s decision-making process: counting positive nuclei for the proportion score and summarizing dominant staining for the intensity score [8]. The segmentation stage yields consistent nuclear instances, addressing challenges specific to PR, including overlapping nuclei, weak boundaries, and heterogeneous morphology [12]. Color deconvolution for isolating DAB produces a monotonic surrogate for biomarker expression. Calibrated thresholds transform this signal into clinically relevant classes (negative, weak, moderate, strong), facilitating a clear and verifiable mapping to Allred components without the need for extensive intensity-labeled data.

Compared to our earlier studies [17,18,19], this work presents a more unified and robust framework by integrating segmentation, classification, and scoring into a single pipeline, with improved evaluation and cross-dataset validation.

This work introduces an integrated PR-IHC scoring pipeline that combines instance-level segmentation, stain-aware classification, and interpretable scoring. Unlike patch-based or end-to-end black-box approaches, the method preserves nucleus-level interpretability, enabling direct mapping between individual nuclei and scoring outcomes. This improves transparency and facilitates clinical validation, especially in PR settings where annotated datasets are limited.

In the literature on ER-IHC, whole-slide pipelines that separate detection, classification, and scoring have demonstrated that automated predictions can align with pathologists at the level of decision-making granularity, including agreement on therapy recommendations at the slide level [5]. The findings expand this paradigm to PR-IHC, demonstrating high agreement at the nucleus level and consistent Allred scores, while also offering transparent intermediate outputs for quality control. Compared to earlier ER-focused systems, the current design prioritizes compact modeling and stain-calibrated decision rules, thereby decreasing the supervision burden and enabling transfer to PR, where curated intensity-annotated datasets are relatively limited [21].

Segmentation quality is the primary source of variance in automated scoring, as both the proportion and intensity components depend on accurate instances. The proposed method demonstrates high precision, recall, and intersection-over-union on PR-IHC test images while maintaining nucleus boundaries in dense or faint areas, providing a solid foundation for subsequent quantification [6]. Previous studies have demonstrated real-time or near-real-time nuclei processing utilizing lightweight decoders, indicating that clinically applicable throughput can be attained [23]. The implementation of a shape-aware instance model incurs a slight computational cost in exchange for enhanced mask integrity on challenging PR slides, representing a beneficial trade-off when the clinical objective is a slide-level score.

In contrast to end-to-end and tile-based slide scorers, the current pipeline maintains a one-to-one correspondence between each nucleus and its class label. This facilitates swift auditing of the positive proportion influencing the Allred sum and allows for targeted recalibration, such as adjusting weak to moderate thresholds, without the need for retraining an extensive network. Systems like IHC-Net demonstrated high accuracy through the integration of deep segmentation and ensemble classifiers [9], while rapid unsupervised pipelines have also exhibited significant agreement under controlled staining conditions [10]. DenseNet-based WSI scorers effectively capture broader context; however, they generally do not provide per-nucleus outputs, which may restrict explainability in proportion-driven scoring [21]. Recent advancements in integrated networks that jointly infer nuclei and staining class have enhanced speed and agreement in ER-IHC [22]. Our contribution is complementary, emphasizing a stain-aware, threshold-calibrated classifier built on a robust instance prior to achieve a balance of accuracy, transparency, and deployment simplicity in PR-IHC.

The observed failure modes are consistent with prior IHC studies [5]. Slides with very low positivity may be affected by minor staining artifacts, leading to slight overestimation of positive nuclei. This can be mitigated through additional quality control steps, such as minimum object size filtering and background stain suppression.

A key strength of the proposed pipeline is its interpretability. Each slide-level decision is supported by nucleus-level outputs, enabling straightforward validation and adjustment. The lightweight and modular design also allows efficient integration into clinical workflows.

This study has several limitations. The dataset is relatively small and derived from a single center, which may affect generalizability. Additionally, calibration was performed using a limited number of ROIs; however, this was intentionally designed to enable lightweight deployment in real-world settings.

Due to practical and therapeutic considerations, a traditional machine-learning and threshold-based approach was purposefully chosen for PR scoring, even though deep learning-based intensity classifiers are frequently seen in ER-IHC literature. First, supervised deep learning intensity models are prone to overfitting and need a significant amount of manual annotation work because PR datasets with trustworthy per-nucleus intensity labels are much fewer than those available for ER. Second, calibrated thresholds generated from stain deconvolved signals offer a straightforward, repeatable, and analytically adequate surrogate for biomarker expression because PR scoring is mostly driven by monotonic DAB intensity rather than intricate textural patterns. Third, pathologists can evaluate and adjust the decision limits without the necessity of retraining extensive networks due to the implementation of stain-aware rules, which ensure that intermediate outputs remain interpretable. This contrasts with end-to-end deep classifiers, which often lack per-nucleus interpretability and require extensive retraining when staining conditions alter. Thus, a traditional machine learning and threshold-based methodology offers an optimal balance of accuracy, interpretability, and deployability for PR-IHC scoring, particularly in resource-constrained or dataset-limited settings.

## 6. Conclusions

We present a comprehensive pipeline for automated evaluation of progesterone receptor (PR) status in breast carcinoma using PR-IHC whole-slide images. The system integrates nuclei segmentation with DAB intensity-based classification and slide-level scoring, forming a complete and interpretable workflow.

The segmentation stage provided a strong foundation for subsequent analysis, achieving high precision, recall, F1-score, and IoU. The classification module accurately assigned nuclei into four intensity categories, with most errors occurring at the weak–moderate boundary. At the slide level, the system showed high agreement with pathologists, achieving 70.0% exact match and 94.0% treatment recommendation concordance.

The framework is distinguished by its integration of instance-level segmentation with a calibrated DAB-intensity classifier, ensuring interpretable and reproducible results. Its lightweight design enables real-time execution without requiring specialized hardware.

Limitations exist. The weak–moderate boundary constitutes the main source of ambiguity at the nucleus level, and slides exhibiting true 0% positivity may be prone to misleading low-level brown signals that artificially increase the proportion score [7]. Further investigation is necessary regarding generalization across institutions and staining protocols. Future work will focus on stain normalization and batch-adaptive calibration, implementing quality-control rules for low-positive floors and background-brown exclusion, and developing hybrid decision support that utilizes a compact neural classifier exclusively for borderline nuclei. The evaluation will be extended to multi-center cohorts, with an exploration of multimarker integration (ER, PR, and HER2) within a unified workflow [6]. Additionally, prospective studies involving pathologists will be investigated to assess time savings, reduction in inter-reader variability, and clinical impact.

This work presents the inaugural whole-slide, end-to-end PR-IHC pipeline, integrating nuclei segmentation, intensity-based classification, and scoring into a coherent, efficient, and clinically relevant tool. The system’s integration of reliability, interpretability, and computational efficiency establishes it as a valuable tool for histopathologists and a robust basis for future multimarker digital pathology applications.

## Figures and Tables

**Table 1:** Proportion Score (P) based on percentage of PR-positive nuclei.

Percentage of Positive Nuclei	Description	Proportion Score (P)
0%	None	0
<1%	Rare	1
1–10%	Low	2
11–33%	Moderate	3
34–66%	High	4
≥67%	Very High	5

**Table 2:** Intensity Score (I) based on dominant staining strength.

Staining Intensity	Intensity Score (I)
None	0
Weak	1
Moderate	2
Strong	3

**Table 3:** Allred score calculation from proportion and intensity scores.

Proportion Score (P)	Intensity Score (I)	Allred Score (P + I)
0–5	0–3	0–8

**Table 4:** Segmentation performance on 50 PR-IHC test images using multiple models. The mean ± standard deviation (SD) is reported for all metrics.

Model	Precision	Recall	F1 Score	IoU
Cellpose (fine-tuned)	0.8882 ± 0.02	0.8215 ± 0.03	0.8535 ± 0.02	0.7445 ± 0.02
Cellpose (baseline)	0.8523 ± 0.03	0.7825 ± 0.03	0.8230 ± 0.03	0.7101 ± 0.03
U-Net	0.8009 ± 0.03	0.7605 ± 0.03	0.7812 ± 0.03	0.6510 ± 0.03
StarDist	0.8601 ± 0.03	0.7902 ± 0.03	0.8305 ± 0.03	0.7203 ± 0.03

**Table 5:** Segmentation performance on 44 ER-IHC test images.

Dataset	Precision	Recall	F1 Score	IoU
ER-IHC (44 images)	0.7200	0.8420	0.7468	0.6018

**Table 6:** Classification performance across four nuclei staining categories on 50 PR-IHC test images.

Class	Precision	Recall	F1 Score
Strong	1.0000 ± 0.00	0.9999 ± 0.00	0.9999 ± 0.00
Moderate	0.9765 ± 0.01	0.9751 ± 0.01	0.9758 ± 0.01
Weak	0.9044 ± 0.02	0.9130 ± 0.02	0.9087 ± 0.02
Negative	0.9118 ± 0.02	0.9095 ± 0.02	0.9106 ± 0.02
Macro Avg.	0.9482 ± 0.01	0.9494 ± 0.01	0.9487 ± 0.01

**Table 7:** Comparison of the proposed DAB-Intensity classifier with common deep learning models.

Model	Precision	Recall	F1 Score	Accuracy
DAB-Intensity Classifier (proposed)	0.95	0.95	0.9487	0.95
Simple CNN Baseline	0.81	0.80	0.805	0.80
ResNet-18 (transfer learning)	0.94	0.94	0.943	0.94
EfficientNet-B0	0.90	0.90	0.902	0.90

**Table 8:** Classification performance on ER-IHC test images.

Metric	Precision	Recall	F1 Score
ER-IHC Classification	0.9380	0.9010	0.9110

**Table 9:** All 50 PR-IHC test slides with nucleus counts and scoring components.

						Scoring		
Image Number	Total Nuclei	Strong	Moderate	Weak	Negative	P Score	I Score	Allred Score
Test image 1	279	176	51	33	19	5	3	8
Test image 2	217	33	41	51	92	4	1	5
Test image 3	148	0	0	0	148	0	0	0
Test image 4	115	0	0	0	115	0	0	0
Test image 5	135	42	38	33	22	5	3	8
Test image 6	132	69	33	13	17	5	3	8
Test image 7	116	4	16	16	80	3	2	5
Test image 8	123	19	14	19	71	4	3	7
Test image 9	123	0	0	0	123	0	0	0
Test image 10	219	32	8	6	173	3	3	6
Test image 11	162	23	15	14	110	3	3	6
Test image 12	82	42	6	7	27	5	3	8
Test image 13	56	0	0	1	55	2	1	3
Test image 14	245	82	50	59	54	5	3	8
Test image 15	243	31	55	76	81	5	1	6
Test image 16	39	0	0	19	20	4	1	5
Test image 17	134	53	18	24	39	5	3	8
Test image 18	155	31	26	56	42	5	1	6
Test image 19	114	20	21	34	39	4	1	5
Test image 20	279	233	36	4	6	5	3	8
Test image 21	129	42	11	25	51	4	3	7
Test image 22	139	30	12	18	79	4	3	7
Test image 23	144	0	0	23	121	3	1	4
Test image 24	100	73	16	1	10	5	3	8
Test image 25	119	1	1	2	115	2	1	3
Test image 26	67	0	0	2	65	2	1	3
Test image 27	176	35	65	38	38	5	2	7
Test image 28	84	0	0	0	84	0	0	0
Test image 29	214	78	22	3	111	4	3	7
Test image 30	40	0	0	1	39	2	1	3
Test image 31	113	3	0	18	92	3	1	4
Test image 32	132	7	15	39	71	4	1	5
Test image 33	92	64	6	1	21	5	3	8
Test image 34	131	47	26	20	38	5	3	8
Test image 35	97	0	0	0	97	0	0	0
Test image 36	130	107	5	5	13	5	3	8
Test image 37	106	98	5	0	3	5	3	8
Test image 38	212	133	43	31	5	5	3	8
Test image 39	84	22	18	20	24	5	3	8
Test image 40	160	0	0	0	160	0	0	0
Test image 41	196	148	27	6	15	5	3	8
Test image 42	189	136	37	6	10	5	3	8
Test image 43	88	0	0	0	88	0	0	0
Test image 44	47	0	0	0	47	0	0	0
Test image 45	138	0	0	0	138	0	0	0
Test image 46	207	92	77	31	7	5	3	8
Test image 47	155	126	17	6	6	5	3	8
Test image 48	78	35	18	14	11	5	3	8
Test image 49	86	75	1	1	9	5	3	8
Test image 50	171	88	14	11	58	5	3	8

**Table 10:** Comparison of breast cancer computational pathology studies based on contributions and reported performance.

Author	Contribution	Result
Liu et al. [28]	Developed a deep segmentation model trained on Ki-67 IHC whole-slide images to delineate invasive carcinoma regions and integrated it with an automated Ki-67 scoring system validated against expert pathologists.	The segmentation achieved over 80% IoU and produced Ki-67 scores that were highly consistent with pathologists.
Lin et al. [29]	Built a mammography-based AI diagnostic pipeline using a large clinical dataset to detect microcalcifications and classify malignant versus benign breasts using deep neural features.	The model reached 0.8124 training and 0.7237 testing accuracy, with malignant sensitivity up to 0.8891.
Li et al. [30]	Proposed a two-stage multiscale CNN with a recurrent attention module for breast cancer histological subtype classification using high-resolution WSI patches.	The system achieved 0.903 patch-level accuracy and 0.846 WSI-level accuracy.
Guo et al. [31]	Introduced an unsupervised multiscale texture-based dynamic learning framework trained on breast cancer histopathology images for image-level classification without manual labels.	Their approach obtained 91.5% accuracy with an F1-score of 91.37%.
Alam et al. [32]	Applied the UNet3+ semantic segmentation architecture to breast cancer histological images to improve delineation of tumor and non-tumor regions for diagnostic support.	The model produced 82.53% segmentation accuracy and 52.57% IoU, with weighted IoU reaching 89.14%.
Lu et al. [33]	Presented BreasTDLUSeg, a hierarchical coarse-to-fine deep learning model designed specifically to segment TDLUs in large-scale WSI datasets containing thousands of annotated patches.	They reported Dice scores of 79.97% and 92.93% across two datasets.
Mahbod et al. [34]	Designed a dual-decoder U-Net architecture trained on H&E nuclei datasets for simultaneous instance segmentation and classification of heterogeneous nuclear structures.	The model achieved panoptic quality scores ranging between 50.8% and 62.1%.
Feng et al. [35]	Developed a multimarker automated scoring system combining segmentation and staining estimation for ER, PR, HER2, and Ki-67 using diverse IHC image sets.	Their reported F1-scores were 0.8553 for ER/PR, 0.8450 for Ki-67, and 0.7962 for HER2.
Hasan et al. [23]	Created a real-time biomarker scoring pipeline using hybrid U-Net, SegNet, and StarDist models to process ER/PR whole-slide images with efficient GPU inference.	The method achieved 90.2% F1-score on a private dataset and 83.3% on a public dataset.
Ahmad Fauzi et al. [5]	Proposed a modular ER Allred scoring system that detects nuclei, classifies staining intensity, and aggregates slide-level scores using a multi-stage CNN pipeline.	Their system achieved 82.5% scoring accuracy on ER-IHC slides.
Ahmad et al. [21]	Performed systematic DenseNet experiments on ER-IHC WSIs, analyzing multiple configurations for tile-level classification leading to final slide-level scoring.	The best configuration reached 81.17% accuracy for ER status prediction.
Lin et al. [22]	Developed a unified encoder–decoder deep network that jointly performs nuclei detection and Allred scoring end-to-end on ER-IHC whole-slide datasets.	The method attained 83.78% scoring accuracy.
This Work	Introduces a fully integrated PR-IHC scoring pipeline combining fine-tuned Cellpose segmentation for PR-specific nuclei with a lightweight, GPU-free DAB-intensity classifier calibrated from stain-deconvolved DAB signals. The design prioritizes morphological accuracy, interpretable decision rules, and low computational requirements, enabling practical deployment in diagnostic workflows. A traditional intensity-based classifier was chosen over deep learning to avoid the need for large, intensity-annotated datasets and ensure stable, reproducible performance under staining variability. Cross-biomarker validation on ER-IHC slides further demonstrates strong generalization and robustness.	The method achieved strong segmentation performance on both biomarkers (PR: F1 = 0.8535, ER: F1 = 0.7468) and high nucleus-level classification accuracy (PR Macro F1 = 0.9487, ER Macro F1 = 0.9110), consistently outperforming deep-learning baselines.

## Data Availability

The PR-IHC image dataset (PR-IHC-40x) used in this study is publicly available via IEEE DataPort. Permalink: https://ieee-dataport.org/documents/pr-ihc-40x-progesterone-receptor-immunohistochemistry-dataset-breast-cancer-diagnosis (accessed on 16 April 2026). DOI: https://dx.doi.org/10.21227/tag3-k180. This dataset represents the primary data used in this study. Additional annotations and derived data supporting the findings are available from the corresponding author upon reasonable request, subject to institutional and ethical approvals. Model code and scripts utilized for nuclei segmentation, classification, and Allred computation can be obtained from the MMU research group upon request for academic, non-commercial purposes.

## Abbreviations

The following abbreviations are used in this manuscript:

ER	Estrogen receptor
PR	Progesterone receptor
HER2	Human epidermal growth factor receptor 2
IHC	Immunohistochemistry
WSI	Whole-slide image
ROI	Region of interest
GT	Ground truth
DAB	3,3′-diaminobenzidine
CNN	Convolutional neural network
IoU	Intersection over Union
F1	F1 score (harmonic mean of precision and recall)
P	Proportion score (Allred component)
I	Intensity score (Allred component)
